# Artificial Intelligence in Decision Support Systems for Type 1 Diabetes

**DOI:** 10.3390/s20113214

**Published:** 2020-06-05

**Authors:** Nichole S. Tyler, Peter G. Jacobs

**Affiliations:** Artificial Intelligence for Medical Systems Lab, Department of Biomedical Engineering, Oregon Health & Science University, Portland, OR 97239, USA; tylern@ohsu.edu

**Keywords:** type 1 diabetes, decision support, Artificial Intelligence, insulin advisor

## Abstract

Type 1 diabetes (T1D) is a chronic health condition resulting from pancreatic beta cell dysfunction and insulin depletion. While automated insulin delivery systems are now available, many people choose to manage insulin delivery manually through insulin pumps or through multiple daily injections. Frequent insulin titrations are needed to adequately manage glucose, however, provider adjustments are typically made every several months. Recent automated decision support systems incorporate artificial intelligence algorithms to deliver personalized recommendations regarding insulin doses and daily behaviors. This paper presents a comprehensive review of computational and artificial intelligence-based decision support systems to manage T1D. Articles were obtained from PubMed, IEEE Xplore, and ScienceDirect databases. No time period restrictions were imposed on the search. After removing off-topic articles and duplicates, 562 articles were left to review. Of those articles, we identified 61 articles for comprehensive review based on algorithm evaluation using real-world human data, in silico trials, or clinical studies. We grouped decision support systems into general categories of (1) those which recommend adjustments to insulin and (2) those which predict and help avoid hypoglycemia. We review the artificial intelligence methods used for each type of decision support system, and discuss the performance and potential applications of these systems.

## 1. Background

Type 1 diabetes (T1D) is a medical condition caused by deficient insulin production and results in dysregulation of blood glucose. Maintaining blood glucose in a target range (70–180 mg/dL) can help to prevent complications related to hyperglycemia (>180 mg/dL) and hypoglycemia (<70 mg/dL), however, this is difficult to achieve for most people with T1D. Existing treatment strategies have evolved over the last 100 years from one-time daily insulin injections, into multiple injections of modern long-acting and rapid-acting insulin formulations, and the automated insulin delivery (AID) systems that are commercially available today [1,2]. While these AID systems are now commercially available, many people choose not to use them for various reasons including cost, inconvenience, issues with form factor, etc. People with T1D may instead prefer to manage their glucose using an insulin pump that delivers fast-acting insulin through continuous subcutaneous insulin infusion (CSII) prior to meals and continuously throughout the day. Or they may instead prefer to use multiple daily injection (MDI) therapy whereby they self-administer fast-acting insulin before meals and long-acting insulin once or twice per day using a needle syringe. MDI therapy continues to be the primary therapy for many people with T1D in the US and worldwide [3].

People who manage their glucose levels using either CSII or MDI therapy must navigate a complicated landscape of heuristic guidelines for the maintenance of basal insulin doses and meal and correction bolus doses. This can be challenging because there are a number of therapy parameters that impact insulin dosing: pre-prandial glucose level, the grams of carbohydrate that they will consume, their insulin sensitivity, their specific insulin-to-carbohydrate ratio, and the current insulin-on-board (IOB). People may also need to consider insulin variations that can occur throughout the day, their current glucose trend, and the activity context under which an insulin dose is being taken (e.g., prior to exercise, during an illness, etc.). This is particularly difficult for people using MDI therapy, as compared to a person using a pump with a bolus calculator, although more recent smart insulin pens have recently made bolus calculation possible for MDI users [4]. Current approaches at heuristic-based guidelines for patients to calculate their insulin dosing can be overwhelming for many people with T1D. In addition, some people may lack the numeracy skills necessary to accurately calculate their insulin prior to meals and throughout the day [5,6]. Ahola et al. found that 64% of patients miscalculate their prandial insulin need, often resulting in repeated hypoglycemia and hyperglycemia [7]. People may also struggle with the challenges of accounting for daily and seasonal variations in their insulin needs, and may wait 12–24 weeks to receive insulin dosage adjustments from their care providers.

The increasing ubiquity of health-based mobile computing and the growing usage of wireless continuous glucose monitors (CGM) [8] has created an opportunity for development of automated decision support systems (DSSs) for people with T1D. A growing number of mobile applications have been developed to provide people with diabetes access to on-demand decision support for their glucose management. Decision support can be provided to a person with T1D either through a health professional who provides the recommendations through a mobile interface, or alternatively directly to the patient using a smart-phone app that automatically generates the recommendations. While some smart-phone-based DSSs have been evaluated in clinical studies [9,10], the outcomes have been mixed with some showing a benefit in terms of improving glycemic outcomes [11,12], while others do not [13,14]. The approaches that have demonstrated the best performance typically involved some form of direct contact between the person with T1D and their care provider [11,12].

Artificial intelligence (AI) is the study of computational approaches that can be used to enable machines to perform intelligent problem solving and accomplish sophisticated tasks. Machine learning is a subset of AI and is specifically the study of how algorithms running on machines can learn and improve performance on a task through past experience and without being specifically instructed to do that task. AI applications that provide decision support to people with T1D have been developed [15,16,17,18,19]. These applications can provide far more frequent insulin dosage adjustment recommendations in between less frequent physician visits. These systems can provide personalized, on-demand insulin bolus calculators. When combined with CGM sensors that provide real-time glucose estimations, AI-based DSS algorithms can also provide personalized hypoglycemia prediction and prevention. Recent studies have demonstrated that AI-recommended insulin dosage adjustments agree with physician opinion with a level of accuracy approaching that of inter-physician agreement [17,19]. Other AI-based systems have demonstrated the ability to reduce hypoglycemia after short-term use [15,16,17,18,19]. In this manuscript, we review AI DSS frameworks that have been evaluated in clinical studies, in silico trials, and retrospective analysis of real-world human data. We discuss emerging trends and future opportunities in AI-based DSSs, which may hold promise for improving glycemic outcomes in people with T1D.

## 2. Common Objectives for Decision Support Systems for People with Type 1 Diabetes

DSSs are designed to help people better manage their diabetes by advising on medication management, alerting to medical complications, providing data visualization, simplifying carbohydrate counting, providing diabetes education, and helping to adjust daily behaviors and lifestyles. In this section, we introduce two types of DSSs: insulin therapy adjustments and hypoglycemia prevention. Adjustments to insulin therapy is one of the most common forms of AI-based decision support. For people using MDI therapy, a DSS can provide guidance on injections of long-acting insulin, and also provide guidance on injections of fast-acting insulin related to meals to help avoid hypo- and hyperglycemic excursions. For people using CSII pump therapy, a DSS can provide guidance on the basal rate of fast-acting insulin for different time windows during the day, as well as boluses related to meals or hypo and hyperglycemic excursions.

The amount of insulin dosed for a meal is proportional to the amount of meal carbohydrates, and is calculated by dividing the estimated carbohydrates by a carbohydrate ratio (g/unit). However, it is quite common for people with T1D and even nutritionists to misestimate the amounts of carbohydrates in a meal, leading to suboptimal estimates of meal insulin. As such, some DSSs covered in this review provide guidance on carbohydrate estimation and also carbohydrate ratio adjustment.

When a person’s glucose is too high, a correction dose of insulin is required. The amount of correction insulin is determined by (1) the person’s current glucose level g(t), (2) their target glucose level g_T_, (3) the current insulin in their body or IOB, and (4) the person’s correction factor (mg/dL/unit). To calculate how much insulin should be dosed when glucose is higher than its target, Equation (1) can be used. Some of the DSSs described in this review provide guidance on correction bolus doses.

(1) $$\mathrm{Advised}\;\mathrm{Correction}\;\mathrm{Dose}=\frac{g\left(t\right)-g_{T}}{\mathrm{correction}\;\mathrm{factor}}-\mathrm{IOB}$$

Hypoglycemia may be particularly challenging for people with T1D. DSSs can provide guidance to help people avoid hypoglycemia in general and also avoid exercise-induced hypoglycemia. Recently developed DSSs are oftentimes closely integrated with CGM sensors, which provide near real-time (typically every 5 min) estimates of interstitial glucose. As a result, these DSSs can utilize CGM to forecast glucose to help people with T1D avoid hypoglycemia. We will discuss both short-term glucose prediction algorithms (i.e., 30–60 min in the future), algorithms that predict glucose during and following exercise, and algorithms that predict glucose overnight, prior to bedtime when hypoglycemia can be particularly dangerous.

## 3. Common Outcome Measures for Assessing Performance of Decision Support Systems

The primary clinical objective of AI-based DSS is to improve glycemic outcomes and prevent medical complications related to diabetes such as hypoglycemia [9]. In this review we focus on AI-based DSS and the measures used to assess DSS performance including accuracy of the underlying algorithms and the clinical impact of using the algorithms.

### 3.1. Clinical Measures of Decision Support Systems

An effective DSS is one which can increase the percent of time that the person with T1D spends in a target glucose range or reduce the percent of time spent in hypoglycemia. The person’s mean glucose is also a measure of glycemic performance whereby a lower mean glucose may mean that they have better glucose control. Hemoglobin A1C (HbA1c) is another commonly used measure that reflects a 3-month estimate of glucose control. Measured HbA1c less than 7% is considered to be within the target range for people with T1D, while values greater than 8.0% are considered very poor glucose control. In addition to these metrics, the high blood glucose index and low blood glucose index (LBGI) by Kovatchev and colleagues has been used to assess the performance of DSSs and AID systems [20].

The benefit of a DSS is typically measured either with respect to the person’s own standard of care or in comparison with a control group population that has not received the DSS intervention. If a DSS is capable of increasing the amount of time (as a percentage of their day) that a person’s glucose is within a target glucose range (70–180 mg/dL) from 50 to 60%, or reducing HbA1c from 8 to 7%, without impacting time in hypoglycemia, that would be considered an impressive performance. A typical commercial AID is capable of increasing absolute time in target range by 5.0% for the Medtronic 670 g [21] and 10% for the Tandem Control-IQ system [2], while reducing the percent time in hypoglycemia from 6.4% to 3.4% and 3.6% to 1.6%, respectively. It remains to be seen whether a DSS can match the level of performance achieved by AID systems.

### 3.2. Measures Used to Evaluate Accuracy of Decision Support Systems

While clinical outcomes can indicate the effectiveness of DSS use, other metrics may be used to assess whether a DSS recommendation is in agreement with recommendations provided by a physician. For a DSS that provides insulin dosage adjustments, the recommendations provided by the DSS can be compared with recommendations provided by a physician. The agreement with a physician can be reported as percent agreement of the DSS recommendations with physician recommendations. The Pearson’s correlation coefficient of the provider recommendation with the DSS recommendation can also be used to evaluate the accuracy of a recommendation.

For DSSs that forecast glucose levels and provide advanced warning of impending hypoglycemia, other traditional metrics are used. A common set of accuracy metrics for algorithms that predict glucose in the future include the root mean squared error (RMSE) and the mean absolute percent error (MAPE). These are typically presented over different prediction horizons (e.g., 30 min to several hours). RMSE and MAPE are defined in equation 2 and equation 3, respectively. The true CGM or glucose value is *Y*, and the model forecasted value is $\hat{Y}$, and N is the number of predicted observations.

(2) $$RMSE=\sqrt{\frac{1}{N}\;\overset{N}{\underset{i=1}{\sum }}{\left({\hat{Y}}_{i}-Y_{i}\right)}^{2}}$$

(3) $$MAPE=\frac{1}{N}\overset{N}{\underset{i=1}{\sum }}\left|\frac{Y_{i}-{\hat{Y}}_{i}}{Y_{i}}\right|\times 100$$

**Figure 1:**
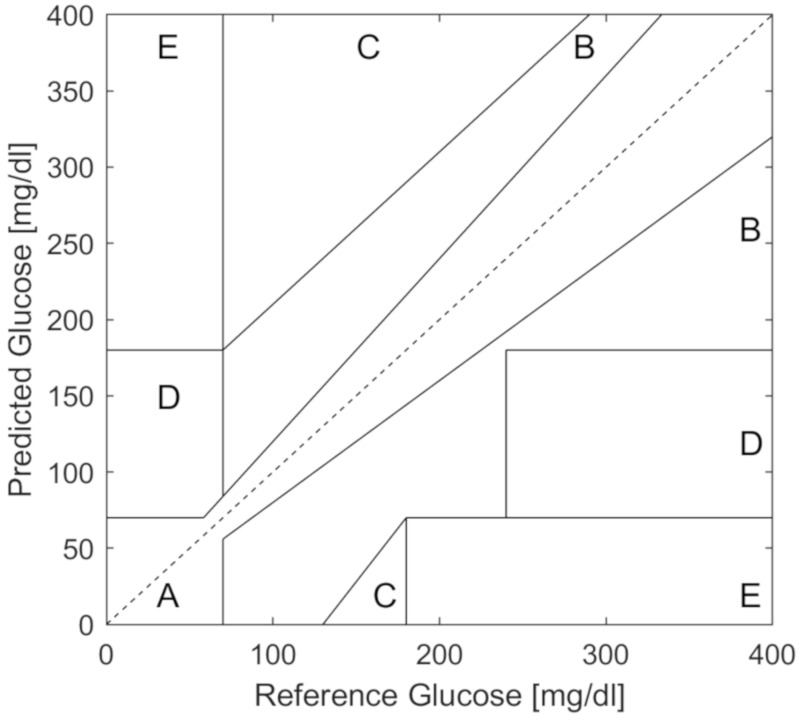
Clarke Error Grid showing predicted (y-axis) vs. reference (x-axis) glucose. The dotted diagonal line shows perfect prediction of glucose. The A region is considered clinically accurate. The B region is considered a clinically safe region of prediction, though not accurate. The C, D and E regions are considered progressively more clinically dangerous regions of prediction.

For quantifying the accuracy of hypoglycemia prediction, traditional measures like sensitivity, specificity, and area under the curve are typically used. In addition, the Clarke error grid [22] and, more recently, the consensus error grid [23] are used to assess the clinical impact of predictions on decisions regarding insulin dosing or carbohydrate treatment. The Clarke error grid (Figure 1) is a plot of true or reference glucose values (x-axis) compared to predicted values (y-axis). The Clarke error grid is divided into clinically relevant regions of A, B, C, D, and E. Regions A and B are considered safe predictions, while C, D and E indicate potentially dangerous predictions where glucose was predicted to be higher than the true value, thereby missing hypoglycemic events or alternatively, glucose predictions were lower than the true value, causing unnecessary carbohydrate consumption. Commercial CGMs typically report 98% percent of values in the A and B regions with no values in the D or E regions as reported from various companies on the US Food and Drug Administration (FDA) web site [24].

## 4. Models and Simulations Used in T1D Decision Support Systems

### 4.1. Physical Models of Glucose-Insulin Dynamics Using Differential Equations

A certain class of the DSSs described in this review relies on physical models of glucose and insulin metabolism dynamics. Such physical models, described below, are comprised of linear and also non-linear differential equations that use compartment models to describe the digestion of carbohydrates, the subcutaneous absorption of injected insulin, and the insulin action effects on glucose metabolism [25]. While these dynamic models can be used to forecast glycemic outcomes [15,26,27], many of these physical models have been developed to simulate the glucose dynamics of T1D populations [28,29] and to design and evaluate DSS algorithms [19].

An interactive diabetes advisor (AIDA) simulator was developed in the early 1990s for use by diabetes educators, clinicians, and their patients [30]. The AIDA simulator consists of a four compartment model describing insulin absorption and elimination, insulin action, carbohydrate absorption, and plasma glucose response. The AIDA software is open-access with an online interface that allows users to input virtual patient features, meal pattern, and insulin doses. The simulator then returns the estimated glucose trends. This system is available as an educational tool [30].

Another physical model of glucose dynamics was published by Hovorka and Wilinska out of Cambridge [25,31]. Hovorka and Wilinska described a non-linear eight-compartment model describing subcutaneous insulin absorption into plasma, the action of insulin on glucose uptake, disposal, and hepatic glucose production, prandial carbohydrate absorption, and plasma glucose levels. Later in 2010, Wilinska et al. [32] outlined an additional two compartment meal model describing the absorption of carbohydrates into plasma glucose, which became a part of the Cambridge Simulator.

The UVA-Padova simulator [28] is one of the most widely used T1D simulators and is described as being accepted as an evaluation tool for artificial pancreas algorithms by the FDA. This system includes multiple compartments to model carbohydrate absorption, glucose availability, insulin transport dynamics and body-wide insulin action, as well as body-wide glucagon production and action. This simulator also includes models for CGM measurement noise, insulin sensitivity variations, subject-specific rescue carbohydrate, and hypoglycemia unawareness.

Other authors have developed models specifically to describe the effects of physical activity on glucose. In 2009, Dalla Man et al., described an exercise model that utilized heart-rate as an input that impacts insulin-independent and insulin-dependent glucose uptake [33]. Roy and Parker modified the Bergman minimal model, a three-compartment model of insulin absorption and activity in plasma and glucose response, to include the effect of maximal oxygen consumption (percent VO_2_) on insulin excretion and hepatic glucose uptake [34].

Resalat et al. [29] modified the eight-compartment model designed by Hovorka and Wilinska et al. [25,31], and created a stochastic virtual patient population by sampling from a distribution of possible insulin sensitivities. Resalat et al. [29] further incorporated an aerobic model of exercise developed by Hernandez-Ordonez et al. [35], describing the impact of physical activity on active muscle mass and insulin sensitivity into their open source simulator. The code for the Resalat et al. simulator is available on Github [36]. In addition to simulating the impact of aerobic exercise on glucose, the simulator includes circadian variations in insulin sensitivity, models for CGM noise, algorithms for simulating response to rescue carbohydrates, administering correction boluses, and dosing behaviors.

### 4.2. Data-Driven Models of Glucose-Insulin Dynamics

While physical models provide a physiologically realistic interpretation of the glucose dynamics, data-driven models have also been used to model glucose-insulin dynamics, especially for shorter horizon (e.g., 30 min) estimations of glucose.

Xie and Wang [37] developed an empirical non-linear autoregressive moving average model with exogenous inputs (NARMAX) that models the bimodal effects of exercise on glucose changes including short-term acute glucose changes and long-term insulin sensitivity changes. This model was trained and evaluated on the Dalla Man et al. [33] model, but not on real-world glucose data. The Dalla Man et al. model is an updated version of the UVA-Padova model [28] that includes a model of physical activity. However, the NARMAX model was shown to effectively model the short and long-term impacts of simulated exercise, achieving a MAPE of 12%, and reported 87% of predictions in Zone A of the Clarke error grid. Further evaluation on real-world human data is needed.

Xie and Wang [38] further evaluated 11 different model approaches to predict glucose using prior glucose, insulin, carbohydrates, and exercise as inputs to the models. They compared less complex autoregressive regression models with exogenous inputs (ARX) with other machine-learning models such as support vector machines (SVM); ElasticNet; gradient-boosted trees; and deep learning models including recurrent neural networks (RNN), long-short-term-memory networks, and temporal convolution networks. While all models had fairly comparable performance in terms of RMSE for predicting short-term glucose dynamics within a 30-min prediction horizon, interestingly, the simpler ARX model had the lowest RMSE at 19.48 mg/dL. However, they also found that the ARX model was more sensitive to spurious noise and tended to under-predict peaks and over-predict minima.

Likewise, adaptive approaches and reinforcement learning are now being utilized to improve glucose predictions. Most recently, He et al. [39] reported that a RNN algorithm with an adaptive learning strategy achieved an 8.46 mg/dL RMSE when predicting glucose 30-min in the future. The evaluation set included people without diabetes, with T1D, and with type 2 diabetes, and further reporting of T1D-specific results are needed.

## 5. Early Approaches at Decision Support System Design

Researchers have been pioneering algorithms used to titrate insulin delivery since the early 1960s [40], however, these approaches were limited by lack of accurate glucose sensor data and short battery life of the devices running the algorithms [41]. Over the years, the algorithms developed from heuristic approaches, to model-based control system approaches, and AI approaches. The goal of the early phases of algorithm development was to build AID systems. More recently, AID and DSS algorithms have become sophisticated and reliable enough to become commercially viable. With the growth of new technologies including smart insulin pens, CGM devices, smart phones, health-related apps, and activity trackers, there is a growing opportunity for the development of DSS algorithms that has not previously been possible.

One of the first DSS algorithms developed was a piece-wise linear algorithm developed by Peterson, Jovanovic, and Chanoch [42,43]. Their algorithm provided basal insulin recommendations using glucose as an input. The algorithm also provided adaptive bolus recommendations and enabled time-dependent dosage profiles. The algorithm was able to reduce the HbA1c in seven people with T1D by 1.6%, from 7.8% to 6.2% after 6 weeks of use, as compared to a 1.5% reduction, from 8.4% to 6.9%, in the standard of care group. One particular set of heuristics published in 1981 by Skylar et al. [44] became a basis for subsequent heuristic approaches to DSSs. These heuristic recommendations were integrated onto portable computers and the authors reported an average Pearson’s correlation coefficient of 0.61 with physician recommendations on real-world human data [45]. The model was further modified and evaluated by Chiarelli and Albisser in a 24-week study in children with T1D. While hypoglycemia increased in both the experimental and standard of care group over the course of the study, the experimental group ended with significantly lower hypoglycemia compared with standard of care at both the 16-week (−1.1%) and 24-week (−1.4%) time points. [46].

Around the same time these heuristic approaches were being developed and tested, model-based DSSs were being harnessed to predict user-specific glycemic responses to insulin regimens. These models were used to approximate a person’s glucose based on a variety of inputs including meals, insulin, and even exercise. In the early 90s, a descriptive study of the Glucoject model was published, which modeled the insulin plasma of different injected formulations for the purposes of treatment replay [47]. Another model approach described by Hauser et al. [48] was developed as an educational tool to help users see the effect of insulin or exercise on their glucose regimens. The authors compared the model predictions of glucose trends to physician-predicted glucose trends, and reported a Pearson’s correlation coefficient of 0.97.

The Karlsburg Diabetes Management System (KADIS) was a model-based DSS that utilized user-entered carbohydrate, insulin, and exercise inputs. Exercise physiology models were not available at the time, and the KADIS algorithm modeled exercise in terms of equivalent insulin units. After fitting a user-specific model from fingerstick glucose, insulin, carbohydrate and exercise data, the model was then used to simulate anticipated meals, planned insulin doses and exercise [49,50]. In 1994, it was marketed as an educational tool for people with T1D and for providers who were less experienced with diabetes management [51]. By 2007, the algorithm had evolved to utilize CGM technologies, and was evaluated in 2007 and 2010 in adults with T1D and T2D. While KADIS-augmented physician support was shown to significantly decrease HbA1c from 7.10 to 6.73% (*p* < 0.01, where *p* is the statistical significance of a hypothesis test) regardless of diabetes type, the system is designed to be utilized by providers once per year to help them adjust their patients’ insulin during visits [52].

DIABETEX was a model-based approach that utilized Bayes decision theory to determine short-acting insulin recommendations. The DIABETEX system was tested on data from 12 adults with T1D and demonstrated a 66% full agreement with endocrinologist-recommended adjustments for meal-related doses, and a 42% full agreement for basal-related doses [53]. The DIABETEX program was later evaluated in children and was reported to improve HbA1c from 5.85% to 5.0%, and reduced the number of measured hypoglycemic events in children from 143 to 58 during a 12-month evaluation period [54].

In the late 1990s, nascent AI and data-driven algorithms were beginning to emerge in the field of diabetes DSSs. Early artificial neural network algorithms used patient demographics and glycemic targets to predict an optimal insulin therapy regimen. Authors reported that the classifier was able to classify 92% of insulin regimens in agreement with health care providers [55,56].

DIAS was a model-based DSS platform pioneered by Carson et al., that utilized probabilistic causal-networks to predict the 24-h glucose profiles for adults with T1D, and then performed model-based replay to determine insulin dosage and carbohydrate intake strategies to minimize hypoglycemia and maximize time-in-range [57]. Evaluation in 20 adults with T1D whereby users were given recommendations by the DIAS system demonstrated equivalent clinical efficacy as compared to recommendations delivered by a diabetes specialist nurse [58].

All of these early algorithms were developed prior to the age of mobile computing. As a result, use of these algorithms required extensive user foresight and planning because they relied on the person with T1D to access a personal computer. While many of these methods proved impractical for real-time DSSs, they set the stage for the explosion of algorithms that occurred when accurate CGM technologies became FDA approved and when mobile computing became ubiquitous.

## 6. More Recent Decision Support Systems

Mobile DSSs are now becoming available for use by people with T1D who use either CSII or MDI and can potentially provide improvements in glycemic outcomes. Existing automated DSSs are designed to provide recommendations to people with T1D regarding insulin doses, anticipated hypoglycemia, and modifications to daily behaviors that may improve their glycemic outcomes. Recent publications on DSS algorithms described advanced control system and traditional AI approaches to deliver personalized recommendations to people with T1D, and have shown promise in providing recommendations that agree with physician opinion. We categorized DSSs into those that provide advice on insulin adjustment and those that provide guidance on hypoglycemia prediction and prevention.

### 6.1. Decision Support Systems for Adjustment of Insulin Therapy

A number of DSSs have recently been developed that may be used to recommend changes to insulin dosing. They generally fall into the category of (1) physiologic model-based algorithms; (2) clustering, or case-based algorithms; (3) heuristic rule-based algorithm; (4) data driven model-based algorithms; and (5) carbohydrate estimations and meal detection algorithms.

#### 6.1.1. Physiologic Model-Based Algorithms

In 2008, Palerm et al. [59] described automated titration of basal insulin pump infusion rates using run-2-run. A run-to-run methodology implies that a model will adapt specific parameters after a run, whereby a run could be considered a day, a meal, an exercise period, an overnight period, etc. If hypoglycemia is observed after one or more runs, the model will adapt and adjust the model parameters to improve future recommendations. Herrero et al.[60] and Toffanin et al.[61] also evaluated run-2-run for adaptation of basal insulin rates, showing an improvement in time in range of 20%[61] and 28% [60], during in silico evaluation in adults in the UVA-Padova simulator. Their run-2-run algorithm was additionally utilized to modify insulin dosage settings. In a study by Zesser et al. in adult participants with T1D, run-2-run bolus adaptation was shown to improve glycemic excursions in the 2 h following meals from 149.1 mg/dL down to 109.4 mg/dL after algorithm convergence [62]. Herrero et al. also utilized run-2-run insulin bolus adaptation, and demonstrated a reduction in hypoglycemia in an in silico study using 10 adults from the UVA-Padova simulator [63].

Physiological models provide a natural basis for insulin dosage adjustment by allowing glucose replay to optimize bolus strategies. In 2008, Wong et al., described a model-based approach for modification of a two-bolus regimen through iterative insulin sensitivity estimations. The authors evaluated this method in an in silico trial using the AIDA simulator and reported a 58–91% reduction in hypoglycemia with use of the adaptive meal bolus calculator, as compared to standard bolus calculator use [64]. Rosales and Garelli described a model-based approach to a meal bolus DSS whereby constrained optimization is used to calculate bolus amount and basal rate during postprandial periods. When evaluated in silico using the UVA-Padova simulator, this approach improved time-in-range from 81.9 to 89.5%, and reduced hypoglycemia from 5.92 to 0.97%, as compared to standard bolus calculator use [65]. Revert et al. also described a method to optimize bolus and basal rate adjustment for different meals using interval analysis [66]. Rosetti et al. evaluated elements of this approach in a clinical study of 12 adults with T1D, and demonstrated that use of the algorithm nominally reduced postprandial area under the glucose curve in the 5 h following a 40 g meal by 103.6 mg*hr/dL, as compared to a standard bolus calculator. However, these results did not translate to a 100 g meal and, therefore, did not achieve statistical significance [67].

Breton et al., also developed a DSS for people utilizing MDI and CSII therapy. This comprehensive DSS included three main algorithms: (1) a model-based insulin-replay for basal recommendations, (2) a Kalman-filter approach for estimation of insulin sensitivity and real-time bolus recommendations, and 3) a logistic regression hypoglycemia prediction algorithm for exercise decision support. During a cross-over study in 24 adults with T1D, participants underwent one 48-h in-patient session per study arm, with standardized meals and aerobic exercise. The authors reported a statistically significant reduction of percent time-in-hypoglycemia following use of the DSS (3.2% [1.3, 4.8] control vs. 0.9% [0.4 2.3] DSS) but did not report significant changes in percent time-in-range [15].

Most recently, Goodwin et al. [26] reported the results of a model based approach, whereby an individual’s glucose dynamics are modeled by fitting a simple glucose model to at-home data to create a digital twin. This personalized model is then modified using stochastic error and disturbances to form an envelope of models. Next, this envelope of models is used to estimate the best bolus shape to be delivered, with specific focus on whether dual-wave bolus, a split bolus, or single bolus should be utilized. This proof of concept study was trained on real-world data from 12 adults, and evaluated 2 years later on two subjects. The results indicated that 54–74% of the real-world data was captured by enveloped predictions. This approach required considerable amounts of subject data with prescribed meal scenarios to train the algorithm, and further evaluation of glycemic outcomes is needed to determine the generalizability of this approach.

#### 6.1.2. Clustering Algorithms

Clustering algorithms are a powerful way to identify glycemic patterns, or groups of similar people with T1D in order to provide user-specific recommendations and glucose predictions. Case-based-reasoning (CBR) DSS algorithms use a database of cases (or examples) of glycemic responses corresponding to specific insulin, meal, exercise, or other inputs. If a new set of observations matches a given case from the database, then the recommendation corresponding to the best-matched case in the database is returned by the algorithm. New cases are added to the database as they are obtained. The k-nearest neighbors approach [68] is a common way of implementing a CBR DSS.

An early CBR-DSS was described in 2002 by Bellazzi et al. [69] as part of the T-IDDM telehealth system. This system was designed to aggregate data from people with T1D, and provide recommendations to physicians who were helping with glucose management. The CBR approach was modified in 2008 by Schwartz et al. [70], who designed a 6-week clinical study in 20 adults with T1D for the purposes of data collection and development of a case-base to be used by physicians. Later in 2010, this system was introduced as the 4 Diabetes Support System [71], but while the authors reported metrics regarding problem identification, we did not find published participant glycemic outcomes.

Herrero et al. [63] described a CBR DSS (named ABC4D) that used an adaptive *run-2-run* algorithm for real-time insulin bolus support. The ABC4D system was evaluated in silico using the UVA-Padova simulator, and demonstrated improved percent time-in-range in 10 virtual adults from 75.2 ± 11.7% to 81.9 ± 13.4% (*p* < 0.05) and a reduction in percent time-in-hypoglycemia from 0.3 ± 0.5% to 0% (*p* = 0.17) after 4 weeks of use. Reddy et al. [18] further evaluated the ABC4D algorithm in a real-world, 6-week clinical study. The authors reported non-significant improvements in percent time-in-range from 55.0% to 60.9%, and also a non-significant reduction in percent time-in-hypoglycemia from 5.0 to 3.6%.

Soon after Reddy et al.’s ABC4D clinical study was published, other approaches to CBR with slight modifications to optimize the adaptive nature of the algorithm were proposed. Similar to the method proposed by Herrero, Torrent-Fontbona and Lopez [72] incorporated “concept drift” into their CBR, effectively replacing old examples in the case-base with newer examples that better reflect the user’s physiologic state. The authors evaluated the algorithm in silico using 11 adults from the UVA-Padova simulator, and reported a time-in-range of 84.0% after 90-days of use with optimal basal settings.

More recently, Tyler and Jacobs [19] published a paper describing a k-nearest neighbors DSS (KNN-DSS) recommender engine that was trained on the OHSU in silico simulator to provide weekly recommendations for updating carbohydrate ratios, correction factors, and basal rates for people with T1D using MDI therapy. The recommendations provided by the KNN-DSS agreed with board-certified endocrinologists 67.9% of the time, which was found to be comparable with inter-physician agreement. When evaluated in silico, percent time in target range increased from 59.5% to 79.8% while maintaining hypoglycemia less than 2%. When evaluated in a small feasibility study on 16 people with T1D whereby physicians provided the recommendations from the engine on a weekly basis to the study participants across 4 weeks, a statistically significant reduction of hypoglycemia events by 43% overnight (*p* = 0.04) and a 25% decrease in hypoglycemia events overall (*p* = 0.051) was observed from the first week of the study to the final week of the study. This KNN-DSS is now being evaluated in a larger clinical trial using a mobile app called DailyDose.

A new system called PEPPER has been proposed by Liu and Herrero [73]. The PEPPER system was designed to analyze user data to deliver real-time hypoglycemia alerts, CBR bolus recommendations, predicted low glucose insulin suspension, and carbohydrate consumption recommendations. In this system, the run-2-run algorithm, which is typically used to recommend new insulin settings, is instead utilized to modify a carbohydrate sensitivity factor for rescue carb recommendations. Six adult participants took part in an 8-week study to evaluate the predictive-low alert system and carbohydrate recommendation components of the PEPPER system, with regular physician decision-support for insulin dosage adjustments. The study found that use of PEPPER resulted in significant decreases in time below 60 mg/dL from 1.8% to 0.7% (*p* = 0.05), and improvements in % time-in-range from 52.8% to 61.3% (*p* = 0.02).

Biagi et al., also described a compositional data analysis k-means clustering algorithm in order to group 24-h glucose profiles. The algorithm was trained using data from six adults with T1D undergoing CSII treatment over 8 weeks. The authors showed that the algorithm returned profile clusters exhibiting high variability, high hypoglycemia, high hyperglycemia, and adequate control. While this algorithm is in the preliminary stages, the approach may be used to categorize glucose profiles for specific activities or behaviors. [74].

#### 6.1.3. Rule-Based Algorithms

Nimri et al. [17] evaluated an AI fuzzy-logic system to adjust insulin pump settings, including basal rate, carbohydrate ratio, and correction factor. The insulin dosage adjustments achieved an agreement with physician recommendations that was comparable to inter-physician agreement. The system was evaluated in a 12-week clinical study whereby adults with T1D underwent engine-augmented physician decision support. The authors reported a reduction in hypoglycemia and improvement in time-in-range after 12 weeks of use, but no significance measures were reported [17].

A more discrete rule-based algorithm called VoiceDiab was proposed by Pankawska et al. [75]. This system utilizes voice recognition for meal entries, and given these verbal recordings of meals, provides estimated nutritional meal content and ultimately recommendations for insulin bolus size and amounts given across time. When evaluated in 12 subjects with T1D during a cross-over study comparing standard bolusing with VoiceDiab bolusing, postprandial glucose excursions were reduced but an increase in hypoglycemia in many subjects was also observed. While this was a heuristic algorithm, this group brings to light two critical areas of consideration for DSSs: exploitation of existing nutritional databases for meal content estimation, and bolus shape optimization.

#### 6.1.4. Other AI Algorithms

Sun et al. [76] described an advisory system for MDI users called ABBA. This system utilizes actor-critic reinforcement learning to retrospectively analyze user data, and supply daily basal insulin dosage suggestions. This system was designed to utilize CGM or self-monitoring blood glucose data (from finger-stick glucose meters) as inputs. An in silico study using 100 adult subjects from the UVA-simulator demonstrated reduction in hypoglycemia from 2.5% to 1.0% while maintaining % time-in-range above 85% after 13 weeks of use.

Srinivasan et al. [77] developed a particle swarm optimization method to determine the optimal meal bolus timing and bolus shapes for meals of different sizes and carbohydrate and fat content using in silico data, and devised a set of heuristics to use for insulin bolusing; however, this method has not been tested yet in humans.

### 6.2. Decision Support Systems for Carbohydrate Estimations and Meal Detection

#### 6.2.1. Computer Vision Algorithms

One of the challenges for people with T1D when dosing meal insulin is estimating the correct amount of carbohydrates within any given meal. While these methods are in their early stages of development, several groups have attempted to leverage smart phone cameras to attempt to automate carbohydrate estimations for people with T1D. The GoCARB system by Anthimopoulos and Mougiakakou [78] used image processing and they trained machine learning algorithms on photographs of food to analyze meal content and provide carbohydrate estimations. The system demonstrated a MAPE of 10% in carbohydrate estimation when evaluated on test images, not used in the training. However, this study was done using a closed set of only 24 meals, with prescribed lighting conditions, making it less applicable to real-world usage. When evaluated by Vasiloglou et al. [79] on a larger set of 54 prescribed meals that again followed specific formulations (e.g., three meal types of food per plate), the system was able to achieve an accuracy of 14.8 g while the estimation by nutritionists was comparable at 14.9 g.

#### 6.2.2. Physiologic Model-Based Algorithms

Many people with T1D fail to announce their meals to DSSs or AID systems and may also simply forget to bolus insulin prior to a meal. Several groups have attempted to utilize CGM patterns to detect meals for the purpose of alerting the person with T1D and reminding them to deliver insulin in case they forget to do so.

Mahmoudi et al. [80] developed a meal-detection algorithm utilizing a Kalman filter, and evaluated it in the UVA-Padova in silico simulator. When their algorithm detected a meal, they used a bolus calculator to administer the insulin determined by the bolus calculator to the virtual subject. In the UVA-Padova simulation, it was demonstrated that the algorithm required 40 min for meal detection, and use of the algorithm improved time-in-range from 53% to 83% for virtual subjects as compared to no meal announcement. This algorithm needs to be evaluated on real-world data. In addition, while this approached utilized an MPC algorithm for use in an AID, evaluation of this meal-detection algorithm and subsequent bolusing may eventually be evaluated for CSII and MDI subjects for use in decision support.

#### 6.2.3. Rule-Based Algorithms

Samadi and Cinar also reported on the design of a fuzzy logic estimated controller for unannounced meal-detection. This algorithm utilized glucose trends and insulin dosed to perform shape identification of glycemic profiles and meal content estimation, resulting in an 87% sensitivity when evaluated in silico, and a 93% sensitivity when evaluated on human clinical data, with a mean time to meal detection of 34.8 min [81]. Though these systems will likely exhibit the best performance within AID or CSII systems where insulin data can be easily tracked, they may also be integrated into MDI-based DSSs to help with meal reminders and carbohydrate estimation.

### 6.3. Decision Support Systems for Hypoglycemia Prediction

While insulin dose adjustment is a critical component of decision support, people with T1D also need to be notified about impending acute glucose changes that could lead to dangerous hypo- or hyperglycemia. Hypoglycemia, if left untreated, can cause coma or death, and even a small number of exposures to extreme hypoglycemic episodes can lead to long term damage to the brain and the heart [82]. In this section, we describe some approaches to preventing hypoglycemia using AI approaches.

Hypoglycemia prediction is largely accomplished through modeling of glucose and CGM trends. Prediction algorithms may be augmented with additional inputs such as insulin data, meal information if available, and physical activity that is either announced or available from fitness tracking devices. An effective DSS would be able to accurately anticipate low glucose levels and notify or alert the person with T1D in advance such that hypoglycemia can be avoided. If the person is using an insulin pump, these predictive algorithms can trigger the insulin pump to stop delivering insulin.

#### 6.3.1. Physiologic Model-Based Algorithms

One Kalman-filter-based approach described by Cameron and Bequette uses 30–70 min prediction horizons. The algorithm has been used to shut off basal insulin if the blood glucose is forecasted to go below 80 mg/dL [27]. This algorithm was evaluated extensively in subsequent clinical trials, and was shown by Calhoun et al. in youth with T1D, and Buckingham et al. in adults with T1D to reduce the number of nights with hypoglycemic events by upwards of 25% in adults [83] and youth [84]. However the overall reported time in hypoglycemia was not reported or found to be significant. By 2017, the algorithm was commercially available in Medtronic pumps under the name SmartGuard and was shown to significantly reduce frequency and duration of hypoglycemia in pediatric participants [85].

#### 6.3.2. Data-Driven Algorithms

In early 2007, Sparacino and Cobelli [86] presented a hypoglycemia prediction model using polynomial models and single-order autoregressive models to predict hypoglycemia with a 30 min prediction horizon window. The models achieved an RMSE of 17–18 mg/dL. In 2010, Perez Gandia et al. [87] further reported on the predictive accuracy of the neural network approach and reported a 17–20 mg/dL RMSE on a 30-min prediction horizon when evaluated on real-world data. Zecchin et al. likewise utilized a neural network prediction strategy and showed through in silico evaluation that hypoglycemia could be significantly reduced through alert-based carbohydrate treatments triggered by the hypoglycemia prediction algorithm [88].

In 2013, Daskalaki and Mougiakakou [89] compared an ARX algorithm, a RNN algorithm, and a fusion approach whereby outputs of both the ARX and RNN were used to improve prediction accuracy. When evaluated on clinical trial data gathered from adults with T1D, the authors reported an RMSE of 18.9 mg/dL using the RNN. The authors also developed an early warning system for hypoglycemia and reported a 100% sensitivity to hypoglycemic events, with a 16.7 min predictive horizon, but with a false alarm rate of 0.8 per day.

#### 6.3.3. Clustering Algorithms

Clustering algorithms, discussed above for CBR approaches to insulin titration, have also been used to improve glucose predictions by Contreras et al. [90]. Specific glycemic profiles were grouped using normalized compression distance clustering, and then cluster-specific grammatical evolution reinforcement learning models were trained. The authors found that the model achieved an RMSE of 4.27 mg/dL for 60-min predictions using in silico data; however, further evaluation on real-world human data is needed as simulator predictions are notoriously far more accurate than those on human data under free-living conditions.

### 6.4. Postprandial Hypoglycemia Avoidance

Recently, groups have developed AI approaches for the purpose of postprandial hypoglycemia avoidance. Montaser et al. [91] developed a seasonal autoregressive integrative moving average with exogenous inputs model of glucose dynamics during the postprandial period. To train the model, data from real-world human subjects were clustered based on exercise type and postprandial glycemic response. CGM data, insulin data, and energy expenditure data were used as inputs to the model. The error reported for the model across all datasets was 6.29 mg/dL for 30 min prediction horizons after the start of meals. It is important to consider that this model was designed and evaluated only on post-meal windows of data. Additionally, it was evaluated on a data set whereby study participants all performed the same exercise and maintained a consistent eating schedule while using closed-loop insulin therapy to manage their glucose levels.

Toffanin et al. [92] studied how data-driven multiple-model predictor (MMP) frameworks could predict post-prandial glycemic patterns across multiple time horizons. While the MMP model predictions were reported to correlate well with human data, the greatest accuracy was observed with morning postprandial glucose responses to meals. This system has not yet been utilized to provide decision support with regards to bolusing.

Oveido et al. [93] developed a predictive model of postprandial hypoglycemia using real-world data from adults with T1D. The authors trained an SVM and demonstrated a 71% specificity for prediction of hypoglycemia in the 4 h period following meals. In silico, Oveido and Vehi [94] demonstrated that these predictive measures could be implemented in a bolus-reduction calculator, and significantly reduced hypoglycemia from 7.6% to 4.68% in the UVA-Padova simulator. Further evaluation using human data has not yet been performed.

Cappon et al., described an extreme gradient-based tree algorithm to predict postprandial glycemic outcomes and modify insulin boluses. The algorithm utilized CGM data, carbohydrate data and insulin data and trained an extreme gradient-based tree algorithm to predict three conditions 6 h after meal consumption: hyperglycemia, in target range, and hypoglycemia. The algorithm was trained and evaluated using UVA-Padova simulated data, and demonstrated a 97% area under the ROC curve accuracy for predicting hypoglycemia following meals. It was demonstrated also within the simulator that use of an algorithm to modify boluses based on predicted probability of hypoglycemia improved time-in-range from 62% to 67%, but no significant reduction in hypoglycemia with use of the bolus modification. This method has not been validated on human data [95].

### 6.5. Nocturnal Hypoglycemia Prediction

Nocturnal hypoglycemia is a dangerous complication of diabetes. Severe episodes may cause coma or death. Prediction and prevention of nocturnal hypoglycemia following normal activity or high physical activity days is a critical area of DSSs.

#### 6.5.1. Linear-Regression Algorithms

One of the earliest algorithms was described by Schiffrin et al. [96] before CGM was available to improve the accuracy of the prediction. Schiffrin et al., utilized linear regressions and logistic regressions to define the probability of hypoglycemia and hyperglycemia given the users glucose at bedtime, and defined a heuristic rule for consuming a carbohydrate before bed if glucose is less than 120 mg/dL. Use of this heuristic was found to reduce the incidence of nighttime hypoglycemia from 13% in control group, down to 0% in the heuristic intervention group.

#### 6.5.2. Support Vector and Other Data-Driven Algorithms

Mosquera-Lopez and Jacobs [97] developed a nocturnal hypoglycemia algorithm trained using CSII data and trained a support vector regression algorithm using data from 124 people (22,804 nights) with T1D from the Tidepool Big Data Donation Dataset [98]. When validated on data from 10 people with T1D on CSII pump therapy across 4 weeks of free-living, and utilizing an announced bedtime of 11 pm and the preceding 15 h of data, the algorithm reported a 94% sensitivity and 72% specificity for predicting nocturnal hypoglycemia. The group also reported a decision-theory approach for selecting the threshold at which to predict low glucose. Similar to Schiffrin et al., they found that adherence to a simple heuristic metric of consuming a carbohydrate if bedtime glucose was less than 149 mg/dL could achieve a 94.1% sensitivity but a lower specificity of 61% specificity for predicted nocturnal hypoglycemia.

Guemes and Herrero [99] utilized an available dataset, OhioT1DM, to compare different machine-learning algorithms to predict the glycemic status of an individual prior to going to bed. After processing input data of CGM, insulin dosed, and self-reported meals, the group developed three classifiers to predict hypoglycemic events (<70 mg/dL), hyperglycemia (>180 mg/dL), and within target range (70–180 mg/dL). The group compared Random forest, artificial neural network, vector machines, linear logistic regressions, and extended tree classifiers. Using an announced bedtime of 11 pm and the preceding 18 h of glycemic data, the authors reported that the SVM achieved the best results, with a sensitivity of 68%, and specificity 71% during cross validation. Hyperglycemia prediction resulted in a 59% sensitivity and 65% sensitivity.

Vehi et al. [100] developed a bimodal hypoglycemia prediction algorithm. An SVM was used to predict postprandial hypoglycemia, while an artificial neural network was developed to predict nocturnal hypoglycemia. The group utilized three databases to train and validate their data, two databases containing data from adults with T1D on insulin pumps, and an additional in silico dataset from the UVA-Padova dataset. The model achieved a 44% sensitivity for predicted nocturnal hypoglycemic events on real-world human data, using a 6 h prediction window.

Bertachi et al. [101] developed a nocturnal hypoglycemia algorithm for MDI utilizing an SVM and multilayer perceptron neural network, trained on 10 adults during a 12-week at-home study. Using data from the 6 hrs preceding sleep, the algorithm achieved a sensitivity of 78% and specificity of 82% for nocturnal hypoglycemia.

### 6.6. Exercise-Induced Hypoglycemia Prediction

Exercise is known to substantially impact glucose levels in people with T1D as covered extensively by Riddell et al. [102]. Steady, moderate intensity, aerobic exercise [103] in particular is known to cause steep drops in glucose in people with T1D. Complicating efforts at decision support on glycemic management during exercise, however, is the fact that other factors can impact glucose dynamics in many different ways. Such factors include the type of exercise, the time of day of exercise, the IOB, the competitive aspect of the exercise, and the person’s level of physical fitness for example. Resistance training and high intensity interval exercise are known to cause less of a drop in glucose during exercise and may even result in increases in glucose, especially when done in the morning in the fasted state, when insulin levels are the lowest [104]. Published guidelines and consensus statement have been developed to assist the management of glucose during exercise [102]. More recently, these guidelines are now being evaluated through clinical trials on high intensity interval exercise to determine how modifications of basal insulin may be helpful in preventing nighttime and mealtime hypoglycemia [105,106]. However, other studies have shown that basal reductions may not significantly prevent hypoglycemia [107]. It has also been shown that exercise-related hypoglycemia can be nominally reduced through use of online educational materials preceding exercise [108], however, these results were not significant. Further development and studies are required to learn how we can best assist people with T1D during and after exercise through DSSs.

While there are several recent publications providing guidance on how and when people with T1D should consider exercising safely, there are not many decision support tools currently available to guide people with T1D during exercise. This represents an opportunity for future AI-based DSS algorithms and apps. If a DSS could predict hypoglycemia during or prior to exercise, it would be able to notify or alert the user in advance so that action could be taken to avoid hypoglycemia. For example, if the person was notified in advance that hypoglycemia was likely, they could consume a carbohydrate prior to exercising to prevent their glucose from dropping too low during exercise. There have been several algorithms published recently that may be used to predict glucose changes or hypoglycemia during exercise.

#### 6.6.1. Linear Regression Algorithms

Ben Brahim et al. [109] use a linear regression to perform a secondary analysis of exercise data collected from 51 people with T1D. They looked at correlations of glucose trends with insulin data, age, total daily insulin requirement, and body weight. The authors determined that the most predictive factors of exercise-related glucose changes were the glucose measured at the start of exercise and the ratio of IOB at the start of exercise to total daily insulin requirement. These features were used in a subsequent hypoglycemia prediction algorithm within an automated DSS. The algorithm provided guidance on carbohydrate consumption prior to exercise session if hypoglycemia was predicted.

#### 6.6.2. Decision Tree Algorithms

Reddy and Jacobs [110] developed a random forest decision tree algorithm that was designed to be used to predict hypoglycemia during exercise. The algorithm was trained and tested using three datasets comprised of CGM data, insulin data, and physical activity data collected from adults with T1D. A random forest model was trained to predict the occurrence of hypoglycemia during either aerobic or resistance exercise, and demonstrated an 86% accuracy. The algorithm was used in a clinical study to automate the shut-off of insulin and bolus glucagon within a bi-hormonal closed-loop system [111].

#### 6.6.3. Data-Driven Models

Hajizadeh and Cinar [112] developed a vector autoregressive model with exogenous inputs (VARX) including CGM, insulin, energy expenditure, and other data collected from a fitness watch. The model’s coefficients were identified in real time and the model was then used for glucose predictions. Real-world data was collected from people with T1D age 19–39 who were participating in a 60-h study of closed-loop AID glucose control. The algorithm yielded a prediction accuracy RMSE of 25.5 mg/dL. This approach was further explored by Hobbs and Cinar [113] to improve glucose predictions during physical activity in adolescents. Data were obtained from adolescents utilizing experimental AID or standard of care CSII devices at a study at ski and snowboarding camps. The group developed an ARX model utilizing carbohydrate, insulin, and heartrate inputs. The group found that utilizing heartrate inputs resulted in better accuracy in terms of a lower RMSE 26.25 mg/dL, as compared to a physical model with a Kalman filter, which had an RMSE of 29.18 mg/dL. However, during the physical activity, larger errors were observed (46.16 mg/dL RMSE). While the authors highlighted the improved prediction accuracy using the VARX model, the results indicate that exercise-specific models may be needed to improve accuracy of predicted glucose-related changes during physical activity.

Romero-Ugalde et al. [114] developed an ARX model to predict glucose dynamics specifically during and after exercise. This group utilized in-clinic data from adults with T1D collected during an exercise study. The inputs to the algorithm included smoothed CGM, insulin, carbohydrate, and activity data. The ARX model that was trained to predict glucose 30, 60, and 120 min after exercise was initiated. They developed both a population model and individualized models specific for each study participant. They found that user-specific models could yield a predictive accuracy of 7.75 mg/dL for a 30-min prediction horizon, which exceeded the accuracy of the population model. While these studies are promising, the authors indicated that the training and evaluation set was comprised of best-case data scenarios, and further evaluation is needed before utilization in a real-time DSS for exercise. However, the results indicate the benefit of using personalized models that can adapt over time to improve accuracy of glucose predictions during exercise.

### 6.7. Exercise-Induced Hypoglycemia Prevention

#### Physiologic Model-Based Algorithms

Fabris et al. [115] discussed the design of a bolus calculator that incorporates an activity-on-board metric, designed to adjust calculated meal boluses given the user’s physical activity history directly preceding the meal. In silico, this was shown to reduce postprandial hypoglycemia in meals following physical activity from 13.4% with standard bolus calculator use to 3.9% with activity-on-board calculator use; however, there are no reports of further evaluation in human studies.

The following year, Fabris and Breton [15] demonstrated that real-time insulin sensitivity estimation for smart bolusing using Kalman filtering could effectively reduce postprandial hypoglycemia in the 4 h period following aerobic exercise. Though this paper did not utilize the activity-on-board algorithm described earlier by Fabris et al., it did demonstrate the utility of insulin sensitivity estimation on glycemic outcomes as described in Breton’s earlier published automated DSS. In a clinical study of this exercise bolus advisor, 15 adults who utilized insulin pumps were enrolled. A 4-week run-in was utilized to gather data and perform subject-specific insulin sensitivity estimation. After the data collection, the subjects underwent two in-clinic exercise sessions and underwent aerobic exercise followed by a standardized dinner. The results showed that the bolus algorithm adjusted for exercise significantly reduced hypoglycemia by nearly 50% in comparison to standard bolus calculations (8.33 vs 14.58, *p* < 0.05), and a significant reduction in postprandial LBGI as compared to standard bolus calculators (1.16 vs 2.86, *p* < 0.05) following exercise [116]. This algorithm shows promise for use in a real-time DSSs following aerobic exercise.

A comprehensive exercise DSS published by Ramkissoon et al. [117] describes use of automated aerobic exercise detection using only CGM data to help reduce hypoglycemia. This algorithm first performs a Kalman filter analysis of historical data collected on non-exercise days to define an activity threshold. Real-time analysis can determine if this threshold has been crossed, at which point the algorithm suggests reduction of basal, reduction of subsequent meal boluses, and a calculated carbohydrate suggestion. Evaluation in silico using the UVA-Padova simulator showed reduction in hypoglycemia from 2.4% to 0.0%, and avoidance of serious hypoglycemia, as compared to AID systems without exercise announcement. In addition, the system effectively reduced the frequency and severity of hypoglycemia in the 2–4 h following aerobic exercise from 2.0% to 0.0%, compared to AID systems with exercise announcement. While these are impressive in silico results, this algorithm has not yet been evaluated in a clinical study.

Further work in the area of preventive carbohydrate consumption was reported by Beneyto and Vehi [118], who developed a feedback proportional-derivative controller to suggest 15 g doses of carbs in real-time if hypoglycemia was predicted by an ARX model. In silico, utilization of this algorithm with simulated aerobic exercise showed a statistically significant decrease in daytime hypoglycemia from 2.2% to 0.9%, and a statistically significant increase in nighttime nadir CGM from 42.8 to 59.2 mg/dL that was significantly different from standard AID use.

Garcia-Tirado and Breton utilized the net-effect replay method to estimate the physiologic disturbance of exercise during in silico studies. This estimate effect was then used as a disturbance input to the MPC controller during announced exercise, showing reductions in hypoglycemia from 3.8% to 0.8% as compared to standard MPC control in silico using the UVA-Padova simulator [119]. Although designed for use within an AID system, similar approaches may be used to estimate short-term and long-term glycemic effects of exercise on real-world human data for use in DSSs. This approach also suggests that exercise may be critical for informing bolus calculators and other components of DSSs in the future.

## 7. Combining Certified Diabetes Education with Decision Support Systems

While many groups strive for the design of fully automated DSSs, combining mobile technology and automated DSSs with a human intervention from a certified diabetes educator (CDE) may lead to optimal improvements in outcomes. Kirwan et al. [12] showed in a randomized trial of 72 people with T1D that weekly feedback from a CDE through text messaging was able to reduce their HbA1C from 9.08 (SD 0.75) to 7.9 (SD 0.75), compared with the control group which showed no significant change. Ideally, someone with T1D may benefit from obtaining regular feedback from a CDE on their insulin dosing strategies without waiting 3–6 months between specialist visits.

CDE systems rely on accurate and innovative ways of displaying their patients’ data that help them provide helpful feedback and guidance. Zhang et al. [120] developed a framework for optimizing the review of glucose data by diabetes educators. Their method automatically aggregates and reconstructs data, allowing for easy display of glucose patterns and insulin dosing behaviors. This system was reported to reflect specialist workflow when reviewed by diabetes care specialists.

While there are many studies that have discussed the impact of diabetes educators for people with T1D, there are far fewer studies evaluating how automated mobile DSSs can best augment CDE-based interventions. A recent meta-analysis [121] has indicated that there needs to be longer, structured studies to evaluate the outcomes of CDE-based support via mobile app or other electronic means.

One such study that integrated a DSS with a CDE intervention involved the Diabeo system. The Diabeo system described by Franc and Charpentier utilizes a heuristic algorithm to advise subjects undergoing CSII and MDI [122]. This mobile Diabeo system included a logbook, bolus calculator, and provides adaptive bolus adjustment for meals, as well as basal adjustment algorithm. Healthcare professionals could also access their patients’ data from a web interface and review glycemic history. Early studies indicated reductions in HbA1C of 0.91% when subjects utilized Diabeo and also received regular decision support from a CDE. The benefits of combining automated mobile decision support and CDE feedback and consultations were described in the TeleDiab I and II studies [11], whereby individuals with type 1 or type 2 diabetes used the Diabeo mobile app with CDE check-ins. The addition of a DSS was reported to have a greater effect on reduction of HbA1c (0.91%) as compared to mobile DSS used alone (0.67%), or CDE intervention alone.

## 8. Potential Future Directions in Decision Support Systems in Type 1 Diabetes

### 8.1. Exercise Decision Support Systems and Exercise as an Adjunct Therapy

This review has identified the need for more extensive tools to help people with T1D better manage their glucose during exercise. There have been relatively few DSS developed that can be used to provide advice to people with T1D across a variety of exercise types, durations, intensities, times of day, insulin loading conditions, and also for people with different fitness levels. The Leona M. and Harry B. Helmsley Charitable trust has recently invested in the funding of a large clinical study called ‘Type 1 Diabetes in Exercise’ (T1-Dexi study) that will yield a large publicly available dataset with time-matched CGM, insulin, food, and exercise data [123]. With new and growing publicly available data sets like this, the area of DSS design in exercise for T1D is likely to be an important future area that will yield many algorithmic advances and new mobile tools in the years ahead.

Exercise as a glycemic intervention is something that almost no one has discussed in the literature. While most of the studies described in this review have focused on how to avoid glycemic excursions caused by exercise, it may be important to consider how exercise can help to achieve glycemic targets. After all, exercise increases glucose uptake and may help reduce postprandial glucose excursions if the correct exercise is done at the right time, at the right intensity, and for the right duration. Xie and Wang [124] explored this concept by designing a non-linear ARMAX model that would recommend the optimal carbohydrate intake, insulin dosage, and target exercise heartrate required to optimize a person’s daily time in glucose target range. In silico evaluation, using the 30 subjects from the UVA-Padova Simulator, indicated use of the recommender system significantly reduced LBGI from 2.57 to 0.42, as compared to standard of care. Clearly, this approach may require considerable planning and foresight, which is not practical for most people with T1D. Nonetheless, this system is unique in that it recommends prophylactic measures that may be taken by a person to exercise in an optimal way to avoid hypoglycemia and maximize glycemic outcomes.

### 8.2. Optimizing Meal Bolus Timing and Other Time-Varying Dosing Parameters

The meal bolus DSSs described in this review have all focused on pre-meal insulin dosing. However, prior work [125] has shown that the timing of insulin delivery both before and following a meal (for CSII users) can help reduce postprandial hyper- and hypoglycemia. For example, for CSII users, a DSS may recommend consuming 50% of the meal insulin bolus prior to eating, and taking the remaining 50% over the next several hours. These bolus insulin suggestions may be adaptive and personalized using run-to-run based outcome measurement assessments to achieve the best glycemic performance possible for a given person.

Adapting the time windows for insulin management parameters like the glucose target, correction factor, and carbohydrate ratio may also show a benefit in improving glycemic outcomes. A person with T1D, whether on CSII or MDI, maintains different glucose targets, carbohydrate ratios and correction factors for specific times of day. Recent positions by the American Diabetes Association have indicated that people with T1D will benefit from different glycemic targets for different times of day and different contexts such as exercise, while sleeping, etc.

Eissa et al. [126] reported a k-means clustering method for determining the optimal time-blocks for bolus calculations. Data from 70 participants with T1D were processed and analyzed using k-means clustering to determine the optimal time windows for insulin dosing parameters. The time-blocks determined by the k-means clustering algorithm were then compared to the time-blocks suggested by clinicians, specialist nurses, and dieticians, and exhibited a 39.1% agreement. This was consistent with the agreement measured between specialists and participants of 36.1%. The algorithm could also identify optimal time blocks for the weekend and also for specific days of the week. This algorithm was not validated in a human study, but simply identifies an interesting method of automating the selection of time blocks for insulin settings based on similar patterns.

However, there has not been a lot of research done in this area to date.

### 8.3. Pregnancy

Use of available technologies such as CSII, CGM, and more recently AID systems [127] have been shown to improve outcomes in pregnant users. Currently, there are no DSSs designed for pregnancy. While the algorithms described here show promise in reducing hypoglycemia and may improve time-in-range in an out-patient setting, these systems require further evaluation in medically complex populations and have not been evaluated in women who are pregnant [128].

### 8.4. Integrating Decision Support Systems with AID

Recent publications by Kovatchev et al. [129] and others have shown that the primary benefit of AID systems is during the overnight period. The primary reason for this is that meals and exercise are very challenging for even an AID system to handle. For example, hypoglycemia that results from exercise may occur even in automated closed-loop systems even when glucagon is delivered in anticipation of exercise [130]. Integrating DSSs with AI may provide a way to improve and optimize AID systems by leveraging patterns observed in glucose and insulin data to help people with T1D make better choices about the settings on the AID systems.

## 9. Conclusions

AI techniques provide a powerful means to address many challenges in diabetes care, and these techniques may be used effectively in the design of DSSs. We have provided a comprehensive review of (1) DSS algorithms that provide insulin dosing recommendations to people using MDI or CSII therapy and (2) DSS forecasting algorithms that provide real-time alerts and notifications with regards to predicted glucose excursions and especially hypoglycemia. While AI is rapidly developing within the field of medical informatics, many of the systems presented in this article utilize traditional machine learning algorithms to adjust insulin therapy or predict hypoglycemia. Cutting-edge machine learning algorithms, such as deep-learning algorithms, have been used in glucose forecasting but have not yet been applied to estimations of insulin dosage adjustments or hypoglycemia prevention.

A common theme on the insulin dosing DSSs which have been evaluated in human studies is that, thus far, they have been effective at reducing glycemic variability and reducing hypoglycemia with short-term use, but they have not yet shown similar improvements in HbA1c, time in range, or mean glucose that AID systems have demonstrated. Like many medical interventions, DSSs require consistent usage to impact clinical outcomes, and so longer studies are required to assess the impact of these DSSs on outcomes such as HbA1c, mean glucose and time-in-range.

One theme from the review of the hypoglycemia prediction algorithms is that there is a wide range of prediction accuracy reported in the literature, and this may be because many of the data sets used for evaluation were either in silico data sets or data sets acquired under prescribed settings within a clinical study. Evaluation of these algorithms with real-world human data under free-living conditions is critical for obtaining a realistic estimate of the algorithm’s accuracy. New big data sets acquired from organizations like Tidepool (San Francisco, CA, USA) and the Tidepool Big Data Donation Data Set [98] are now becoming available that include real-world, free-living CGM, insulin, and exercise data from people using CSII and more recently AID systems. The Leona M. and Harry B. Helmsley Charitable Trust is sponsoring a large study to generate time-matched CGM, insulin, nutrition, and exercise data in 600 people with T1D [123]. These data sets should be leveraged so that algorithms can be evaluated, compared, and benchmarked on a common, real-world data set.

Lastly, many of the insulin-dosing DSS algorithms and glucose prediction DSS algorithms reviewed here included only an in silico evaluation, making it challenging to assess how well the systems will ultimately perform in actual humans. While the in silico simulators have been absolutely critical and transformative in the design and preliminary evaluation of DSSs and AID systems, human testing should be a goal for any group that is serious about having their DSS algorithm translated to use by people with diabetes.

## 10. Materials and Methods

We performed a comprehensive search of PUBMED, IEEE Xplore, and ScienceDirect utilizing combinations for the following terms: “exercise OR Physical”, “hypoglycemia”, “type 1 diabetes”, “prediction”, “decision support”, “insulin adjustment”, “insulin management”, “decision algorithm”, “exercise adjustment”, and “carbohydrate intake”. No time span was imposed on these papers. After removing duplicate articles, we were left with 562 primary research articles, conference articles, and book chapters. Of these 562 items, we prioritized approaches that include validation via human pilot studies or clinical trials, and secondly considered novel approaches that evaluated their algorithms through in silico trials.

We did not include papers that described a system framework without algorithm descriptions, or without preliminary results on in silico or human data. While many glucose-prediction approaches were returned by our search, we only include the most recent state-of-the-art approaches. We likewise excluded AID and MPC control algorithm papers that did not relate to DSSs. All papers reviewed are included in Table S1.

## Supplementary Materials

The following are available online at https://www.mdpi.com/1424-8220/20/11/3214/s1, Table S1: Summary of decision support strategies evaluated in this review.
