# Supplementary material for: Artificial Intelligence in Decision Support Systems for Type 1 Diabetes
Source: Sensors (Basel). 2020 Jun 5;20(11):3214. doi: 10.3390/s20113214 (PMC7308977; doi:10.3390/s20113214)
Supplement: Supplementary file 1 [file sensors-20-03214-s001.pdf]

Supplementary

Table S1. Summary of decision support strategies evaluated in this review.

Citation	Purpose of Decision Support	Algorithm	Evaluation strategy	Study type / Dataset	Reported Outcome
Bellazzi et al	Insulin dose adjustment	Case-based reasoning	Real-world use	Clinical Study	% HbA1c
Schwartz et al	Insulin dose adjustment<br>Carbohydrate intake	Case-based reasoning	Retrospective analysis of real-world data	Data collection<br>Secondary data analysis	Accuracy of identified cases
Herrero et al	Insulin dose adjustments	Case-based reasoning<br>Run-2-Run	Simulated use	*In silico* clinical study: UVA-Padova	% Time-in-range<br>% time-in-hypoglycemia
Reddy et al	Insulin dose adjustment	Case-based reasoning<br>Run-2-Run	Real-world use	Clinical Study	% Time-in-range<br>% time-in-hypoglycemia
Torrent Fontbona et al	Insulin dose adjustment	Case-based reasoning<br>Concept drift	Simulated use	*In silico* clinical study: UVA-Padova	% Time-in-range<br>% time-in-hypoglycemia
Liu et al	Hypoglycemia alarm<br>Carbohydrate intake	Run-2-Run	Real-world use	Clinical study	% Time-in-range<br>% time-in-hypoglycemia
Tyler et al	Insulin dose adjustment	K-nearest-neighbors<br>Model-based bolus adjustment<br>Heuristic quality-control	Real-world use	Clinical study	% Time-in-range<br>% time-in-hypoglycemia
Biagi et al.	Glycemic Pattern Identification	K-means clustering	Retrospective analysis of real-world data	Secondary data analysis	Glycemic profiles
Nimri et al	Insulin dose adjustment	Rule-based fuzzy logic	Real-world use	Clinical study	% Time-in-range<br>% time-in-hypoglycemia
Pankawska et al	Insulin dose adjustment<br>Carbohydrate estimation	Voice recognition<br>Rule-based heuristics	Real-world use	Clinical study	% Time-in-range

Palerm et al	Basal insulin dose adjustment	Run-2-Run	Simulated use	Proof of concept	Time to convergence
Herrero et al	Basal insulin dose adjustment	Run-2-Run	Simulated use	*In silico* clinical study: UVA-Padova	% Time-in-range<br>% time-in-hypoglycemia
Toffanin et al	Basal insulin dose adjustment	Run-2-Run	Simulated use	*In silico* clinical study: UVA-Padova	% Time-in-range<br>% time-in-hypoglycemia
Zisser et al	Bolus insulin dose adjustment	Run-2-Run	Real-world Use	Clinical Study	Mean glucose preceding and following meals
Wong et al	Insulin dose adjustment	Model-based simulated replay	Simulated use	*In silico* clinical study: AIDA simulator	% HbA1C<br>% time-in-hypoglycemia
Rosales et al	Insulin bolus dose and shape	Constrained optimization	Simulated use	*In silico* clinical study: UVA-Padova	% Time-in-range<br>% time-in-hypoglycemia
Revert et al<br>Rosetti et al	Insulin bolus dose and shape	Interval analysis	Real-world Use	Clinical Study	Postprandial AUC
Breton et al	Insulin dose adjustment<br>Exercise hypoglycemia prevention	Kalman-filter state estimation<br>Model-based simulated replay<br>Logistic regression	Real-world use	Clinical study	% Time-in-range<br>% time-in-hypoglycemia
Goodwin et al	Insulin bolus dose and shape	Model-based forecasting	Retrospective analysis of real-world data	Data collection<br>Secondary data analysis	% of subject data lying within the prediction envelope
Sun et al	Insulin dose adjustment	Actor-critic reinforcement learning	Simulated use	*In silico* clinical study: UVA-Padova	% Time-in-range<br>% time-in-hypoglycemia
Perez-Gandia et al	Insulin dose adjustment<br>Carbohydrate intake	Artificial neural network	Real-world use	Clinical Study	Kovatchev's risk index
Srinivasan et al	Insulin bolus dose and shape	Particle swarm optimization	Simulated use	Proof of concept study, *In silico* clinical study: UVA-Padova	% Time-in-range<br>% time-in-hypoglycemia
Anthimopoulos et al	Carbohydrate Estimation	Computer vision	Real-world use	Proof of concept study	Mean absolute error

Vasiloglou et al					
Mahmoudi et al	Missed meal detection	Kalman-filter state estimation	Simulated use	*In silico* clinical study: UVA-Padova	% Time-in-range<br>% time-in-hypoglycemia
Samadi et al	Missed meal detection	Rule-based fuzzy logic	Retrospective analysis of real-world data	Secondary data analysis	% Sensitivity<br>% false positive rate for meal detection
Zhang et al	Glycemic pattern identification	Heirarchical task abstraction	Retrospective analysis of real-world data	Qualitative proof-of-concept	Physician feedback
Charpentier et al	Insulin dose adjustment	Rule-based heuristics<br>Clinical diabetes educator	Real-world use	Clinical trial	% HbA1c
Cameron et al<br>Calhoun et al<br>Buckingham et al<br>Biester et al	Basal insulin suspension	Kalman-filter state estimation	Real-world use	Clinical study	% HbA1c<br>% time-in-hypoglycemia
Sparacino et al	Glucose forecasting and hypoglycemia prediction	Data-driven ARX	Retrospective analysis of simulated data	*In silico* clinical study: UVA-Padova	RMSE of forecasted glucose
Perez Gandia et al	Glucose forecasting and hypoglycemia prediction	Artificial neural network	Retrospective analysis of real-world data	Secondary data analysis	RMSE of forecasted glucose
Zecchin et al	Glucose forecasting and hypoglycemia prediction	Artificial neural network	Simulated use	*In silico* clinical study: UVA-Padova	% Time in hypoglycemi
Daskalaki et al	Glucose forecasting and hypoglycemia prediction	Data-driven cARX<br>Recurrent neural network	Retrospective analysis of real-world data	Secondary data analysis	RMSE of forecasted glucose<br>% Sensitivity for hypoglycemia prediction
Contrares et al	Glucose forecasting	Clustered grammatical evolution<br>Reinforcement learning	Retrospective analysis of simulated data	*In silico* clinical study: UVA-Padova	RMSE of forecasted glucose

Montaser et al	Postprandial hypoglycemia prediction	Data-driven ARIMAX	Retrospective analysis of real-world data	Secondary data analysis	RMSE of forecasted glucose
Toffanin et al	Postprandial hypoglycemia prediction	Data-driven state-space	Retrospective analysis of real-world data	Secondary data analysis	FIT and Coefficient of determination of forecasted glucose
Oveido et al<br>Oveido et al	Postprandial hypoglycemia prediction<br>Insulin bolus adjustment	Support vector regression	Retrospective analysis of real-world data<br>Simulated use	*In silico* clinical study: UVA-Padova	% Sensitivity for hypoglycemia prediction<br>% time-in-hypoglycemia following meals
Cappon et al	Postprandial hypoglycemia prediction<br>Insulin bolus adjustment	Xtreme gradient-boosted tree	Simulated use	*In silico* clinical study: UVA-Padova	AUROC<br>% time in range<br>% time in hypoglycemia
Schiffrin et al	Nocturnal hypoglycemia prevention, Carbohydrate intake	Linear regression<br>Decision theory	Real-world use	Clinical study	Incidence of hypoglycemia<br>% HbA1c
Mosquera-Lopez et al	Nocturnal hypoglycemia prediction and prevention	Support vector regression<br>Decision theory	Retrospective analysis of real-world data<br>Simulated use	Secondary data analysis *In silico* clinical study: OHSU T1D	% Sensitivity and % Specificity of predicted nocturnal hypoglycemia
Guemes et al	Nocturnal hypoglycemia prediction	Support vector regression	Retrospective analysis of real-world data	Secondary data analysis	% Sensitivity and % Specificity of predicted nocturnal hypoglycemia
Vehi et al	Nocturnal hypoglycemia prediction	Support vector regression<br>Artificial neural network	Retrospective analysis of real-world and simulated data	Secondary data analysis	% Sensitivity and % Specificity of predicted nocturnal hypoglycemia
Bertachi et al	Nocturnal hypoglycemia prediction	Support vector regression,	Retrospective analysis of real-world data	Secondary data analysis	% Sensitivity and % Specificity of predicted nocturnal hypoglycemia

		Multilayer perceptron neural network			
Fabris et al	Exercise hypoglycemia prevention, insulin bolus adjustment	Model-based activity on board adjustment	Simulated use	*In silico* clinical study: UVA-Padova	% time in range<br>% time in hypoglycemia
Fabris et al	Exercise hypoglycemia prevention, insulin bolus adjustment	Kalman-filter state estimation	Real-world use	Clinical study	% time in range<br>% time in hypoglycemia
Ramkissoon et al	Exercise hypoglycemia prevention, insulin dose adjustment, carbohydrate intake	Kalman-filter state estimation	Simulated use	*In silico* clinical study: UVA-Padova	% time in range<br>% time in hypoglycemia
Beneyto et al	Exercise hypoglycemia prevention, carbohydrate intake	Proportional derivative controller	Simulated use	*In silico* clinical study: UVA-Padova	% time in range<br>% time in hypoglycemia
Garcia-Tirado et al	Exercise hypoglycemia prevention	Model-based characterization of exercise	Simulated use	*In silico* clinical study: UVA-Padova	% time in range<br>% time in hypoglycemia
Ben Brahim et al	Exercise-related glucose forecasting	Linear regression	Retrospective analysis of real-world data	Secondary data analysis	Pearson's correlation, Predictive features
Hayeri	Exercise-related glucose forecasting	Gradient boosted decision trees<br>Support vector regression	Retrospective analysis of real-world data	Secondary data analysis	Clarke error grid
Reddy et al	Exercise hypoglycemia prediction	Random forest decision tree	Retrospective analysis of real-world data	Secondary data analysis	% Sensitivity and % Specificity of predicted exercise hypoglycemia
Hajizadeh et al<br>Hobbs et al	Exercise-related glucose forecasting	Data-driven ARX	Retrospective analysis of real-world data	Secondary data analysis	RMSE of forecasted glucose
Romero-Ugalde et al	Exercise-related glucose forecasting	Data-driven ARX	Retrospective analysis of real-world data	Secondary data analysis	RMSE of forecasted glucose

Eissa et al	Adjustment of time-blocks used for insulin dosing	K-means clustering	Retrospective analysis of real-world data	Data-collection Secondary data analysis	Agreement with specialists
Avila et al	Adjustment of glycemic target	Decision tree, Recommender system	Retrospective analysis of simulated data	Secondary data analysis	RMSE of predicted glycemic variability
